# Simulations with Australian dragon lizards suggest movement-based signal effectiveness is dependent on display structure and environmental conditions

**DOI:** 10.1038/s41598-021-85793-3

**Published:** 2021-03-18

**Authors:** Xue Bian, Angela Pinilla, Tom Chandler, Richard Peters

**Affiliations:** 1 Animal Behaviour Group, Department of Ecology, Environment and Evolution, La Trobe University, Melbourne, VIC Australia; 2 Faculty of Information Technology, Monash University, Caulfield East, VIC, Australia

**Keywords:** Animal behaviour, Behavioural ecology

## Abstract

Habitat-specific characteristics can affect signal transmission such that different habitats dictate the optimal signal. One way to examine how the environment influences signals is by comparing changes in signal effectiveness in different habitats. Examinations of signal effectiveness between different habitats has helped to explain signal divergence/convergence between populations and species using acoustic and colour signals. Although previous research has provided evidence for local adaptations and signal divergence in many species of lizards, comparative studies in movement-based signals are rare due to technical difficulties in quantifying movements in nature and ethical restrictions in translocating animals between habitats. We demonstrate herein that these issues can be addressed using 3D animations, and compared the relative performance of the displays of four Australian lizard species in the habitats of each species under varying environmental conditions. Our simulations show that habitats differentially affect signal performance, and an interaction between display and habitat structure. Interestingly, our results are consistent with the hypothesis that the signal adapted to the noisier environment does not show an advantage in signal effectiveness, but the noisy habitat was detrimental to the performance of all displays. Our study is one of the first studies for movement-based signals that directly compares signal performance in multiple habitats, and our approach has laid the foundation for future investigations in motion ecology that have been intractable to conventional research methods.

## Introduction

Animal signals evolve to be effective in the environment in which they are emitted^1–3^, and therefore the diversity we see in animal communication systems is partially driven by the environment in which the animal lives^4,5^. An effective signal requires reliable detection by an intended receiver following transmission through ecologically complex environments^1^. It is evident that environmental structures and habitat-specific characteristics can affect signal transmission^1,4,6,7^, such that habitat characteristics contribute to shaping the optimal signal necessary for reliable detection^8^. Dynamically changing abiotic and biotic noise in the environment also interferes with effective communication between individuals, and animals are required to produce signals that could compete with these irrelevant sensory inputs^3,9,10^. Many animals modify their signalling behaviour in response to changing conditions^11–15^, such that these moment-to-moment adjustments to signalling strategies represent a form of behavioural plasticity to maintain signal effectiveness^16,17^. Clearly, habitat-specific transmission properties are important in determining the optimal structure of a signal, and it is therefore imperative that we quantify signal effectiveness in the context of the relevant noise landscape to understand in more detail the evolution of animal communication strategies.

One way to examine how the environment influences signals is by comparing changes in signal effectiveness in different habitats. Earlier research into quantifying acoustic characteristics of different habitat types provided the foundation for understanding diversity in the physical structure of avian songs^6,18^. Cross-habitat examination of signal effectiveness between different habitats also helped to explain intraspecific divergence of calling behaviours in anuran species^19^. Similarly, by contrasting signals in different habitats, signal divergence was described for lizard populations using colour-based signals^20^, as well as closely related insect species with seismic communication strategies^21^. Structural differences in movement-based signals have also been observed in many lizard species occupying both similar and distinctive habitats^17,22–24^. Bloch and Irschick^25^ found temporal differences in the display sequences of the green anole, *Anolis carolinensis*, suggesting population divergence due to population density and habitat use. Similarly, structural differences in the core display of the Jacky dragon, *Amphibolurus muricatus*, between three different populations might be a consequence of behavioural plasticity in response to variation in habitat structure^26^. Clearly, movement-based signals also show variation consistent with local adaptation explanations, but detailed cross-habitat comparisons of signal structure has rarely been demonstrated. Aside from the technical difficulties associated with quantifying the environmental conditions and animal displays^27^, there are important legislative and ethical restrictions that prevent translocating animals between habitats that they do not naturally inhabit.

Plant movements are the primary source of motion noise that affects movement-based signal communication^14,28–31^. However, the motion noise landscape is difficult to examine in a controlled, systematic way, as it differs vastly from one microhabitat to another as a result of the interaction between variable plant characteristics, habitat topography and microhabitat structure^32^. To our knowledge, the recent work by Ramos and Peters^33,34^ represents the only attempts to consider the relative effectiveness of movement-based signals of lizards in multiple habitats. In the first of these studies, evidence is presented for local adaptation, showing that two sympatric lizard species who use different movements in their displays, nonetheless produced similar motion speeds^33^. In the second paper, the authors showed structural differences between allopatric populations of the same species^34^. In both studies, habitat structure was considered to be the guiding force leading to convergence or divergence in structure respectively. However, the approach taken in both of these field-based studies was to film displays in local environments, and then the movement of surrounding plants at a separate time, in a controlled manner, and to then contrast resultant motion speed data^35^. Although these are novel and informative insights, we have developed new techniques based around sophisticated three-dimensional (3D) animations that affords us the opportunity to consider movement-based signals *embedded* in noise^27^.

The aim of the present study was to consider directly the relative effectiveness of the movement-based displays of multiple lizard species in multiple habitats using 3D animation. We reused the subject of earlier work^27,36^, the Jacky lizard, *A. muricatus*, and created three additional model lizards and their respective habitats: the long-nosed dragon, *Gowidon longirostris*, the Mallee dragon, *Ctenophorus fordi*, and the tawny dragon, *C. decresii* (Figs. S1, Fig. S2). In addition to quite distinct signals, these four species also occupy different habitat types, from densely vegetated coastal heath environments to sparsely vegetated rocky outcrops and semi-arid Mallee woodland with spinifiex understories (Fig. S2). The associated noise landscape in these habitats also varies as a result of vegetation density and plant species present. Signals adapted to a noisier environment tend to perform better when placed in a less noisy environment^33,34^. Therefore, we predicted that the signal of *A. muricatus* would perform better in the other three habitats as it occupies a densely vegetated and noisy environment. Similarly, we would also expect the habitat of *A. muricatus* to have the greatest influence on signal performance. The habitats of *G. longirostris* and *C. decresii* are very similar, so we predicted that these will influence signal performance of all species in a similar manner, and that their displays would perform equivalently. *Ctenophorus fordi*, on the other hand, would produce relatively weak signals as this species uses the simplest motor pattern compared to the other three species.

Habitat structure is a crucial determinant of signal structure across species and modalities and here we extend this literature to include the movement-displays of lizards from different habitats. The diverse habitats are each inhabited by only one of the focal species, and experimental translocation of absent species would not be permissible. While we have simulated these circumstances, 3D animation technology is sufficiently advanced that these are excellent proxies for filming the same circumstances in nature (if it were possible). Our animations are detailed and founded on the natural systems they represent.

We now have a way to obtain the input, but we also needed a way to determine the relative effectiveness of the movement-based displays in motion noise. Determining what attracts attention in complex scenes is not trivial^37^. We can certainly use biologically inspired motion vision algorithms to quantify movement in image sequences^10,38^. These algorithms can tell us where movement occurs, as well as the direction and magnitude of that movement, but provides no insight into which movements should attract attention. For the present purposes, we needed to predict where attention is drawn given multiple sources of movement. Research into selective attention across multiple disciplines has considered how biological systems solve this problem and one avenue of investigation has suggested that attention could be directed by bottom-up processes that rely on sensory-driven differences^39^. Attention is drawn to specific cues or features that are distinct, and it is this distinctive quality that makes them more *salient*. Biologically inspired computational models of attention have incorporated the notion of saliency to create saliency maps that combine multiple features such as colour, orientation, intensity, flicker and motion^40,41^. Internal competition through biologically inspired dynamic neural networks of integrate-and-fire neurons are used to determine the ‘winning’ location (see^42^), and the predicted focus of attention^40^. Saliency analysis provides a computational approach that suits our needs. It is biologically inspired, incorporating knowledge of the visual motion processing systems of primates to insects, and yields predictions on the relative salience of different parts of the scene. Nonetheless, although widely used, the empirical data in support of the models is limited to relatively simple tasks by humans, and there is ongoing discussion about its application^43^. Consequently, we apply it here with caution and do not assume it represents the processes underlying motion segmentation and selective attention in lizards.

Sophisticated 3D animations have provided us ‘footage’ that cannot be obtained in nature, while our analyses of image sequences are informative but not specific to lizard sensory and brain properties. We equate high salience with effective movements but recognise that empirical data supporting this is lacking. Nevertheless, our entirely novel simulations provide evidence to support the hypothesis that signal effectiveness is dependent on signal structure and the environmental conditions in which they are emitted.

## Methods

### Study species

**Figure 1:**
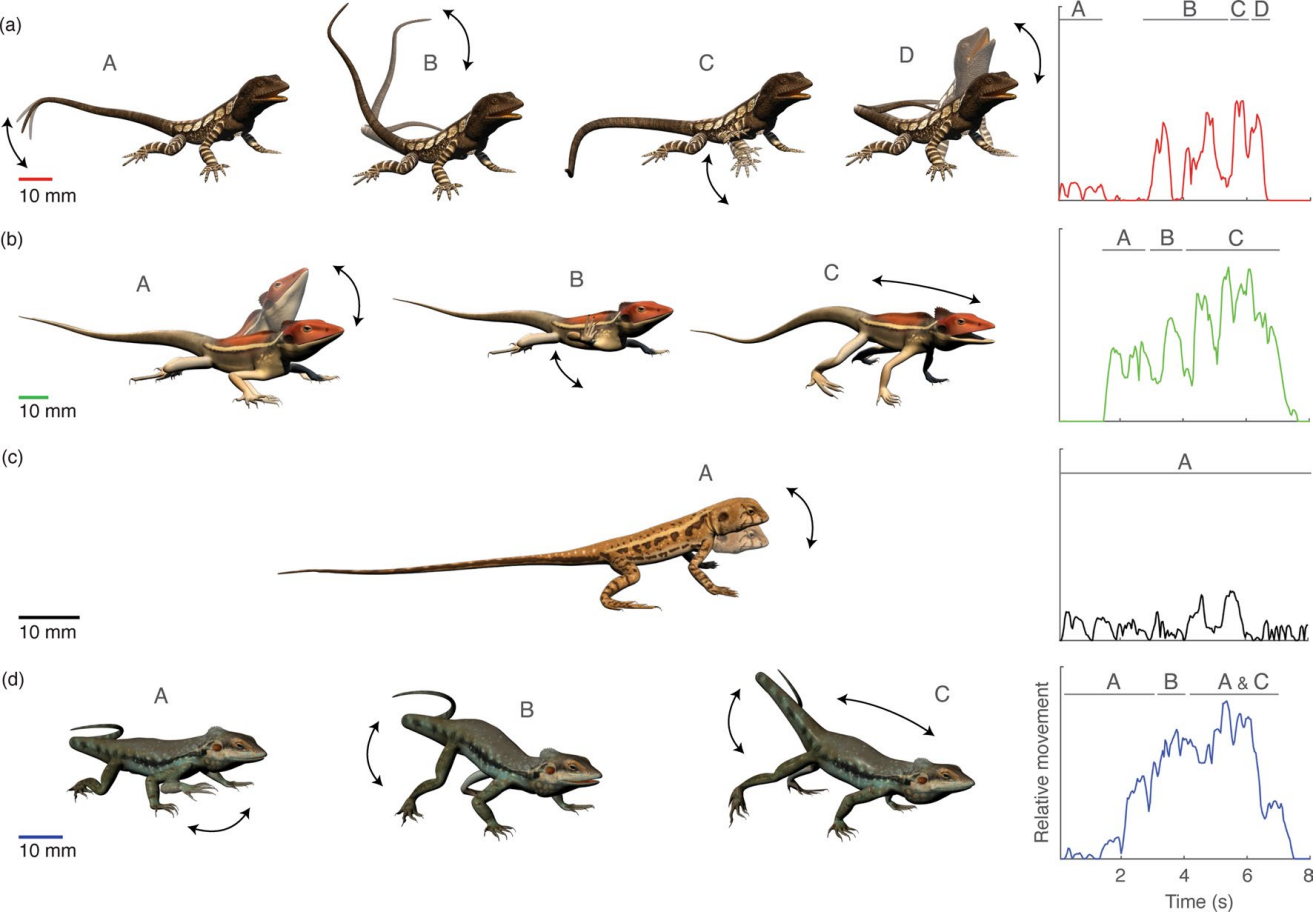
Images depicting the display sequences of the four lizard species used in the present study (left) accompanied by the respective temporal structure profile graph (right; see text for details). In each image set, black arrows indicate the direction of movement. Motions such as head-bobs, limb waves and tail-flicks are usually repeated for several cycles. (**a**) The display sequence of *A. muricatus* starts with tail-flicks (A, B), followed by limb waves (C) and finishes with a whole body movement centred on a push-up (D). The temporal structure graph indicates a relatively intermittent movement throughout the sequence. (**b**) The display sequence of *G. longirostris* comprises a series of head bobbing (A) and limb waving (B), the final push up is followed by a forward body thrust/rise (C). The temporal structure graph indicates relatively constant movement but with several rapid changes in movements throughout the whole sequence. (**c**) The display of *C. fordi* is comparatively simple, and consists of rapid movement of the head up and down (head-bobbing, A). The temporal structure graph indicates a relatively continuous movement throughout the sequence. (**d**) The display sequence of *C. decresii* comprises with several limb waves (A) and a distinct raising of the tail with a coil at the distal end of the tail (B), it finishes with a forward body thrust with tail coiled (C). The temporal structure graph indicates relatively constant movement, featuring a gradual increase in the amount of body movement. Scale bars are provided for each species. Lizard images were created by the authors using Autodesk Maya 2015 (https://www.autodesk.com.au/products/maya/).

*Amphibolurus muricatus* (snout-vent length, SVL, 122 mm^44^) is a well-known species that has featured in many physiology, ecology and visual communication studies^14,35–48^. The display of *A. muricatus* begins with tail-flicking, followed by limb movements and a push-up, sometimes accompanied by throat extensions^49,50^ (Fig. 1a). There are two parts to the tail-flick sequence: an initial intermittent movement at the tip of the tail followed by a continuous and more rigorous movement of the whole tail. The tail-flicks serve as an introductory component to the rest of the display and are used to attract the attention of the intruder^31,51^. *Amphibolurus muricatus* inhabits woodlands and coastal heath of southeast Australia and are often seen perched on fallen timber^44^. The simulated *A. muricatus* habitat was based on sites around the Croajingolong National Park, Victoria, where the habitat is densely vegetated with a complex understory of tall grasses and shrubs (Fig. S3a).

In contrast to *A. muricatus*, the displays of *Ctenophorus fordi,* are much reduced in complexity, and almost exclusively comprised of head bobbing (Fig. 3c). *Ctenophorus fordi* are found in south-east Western Australia to western Victoria and New South Wales^44^. The species is smaller in size (SVL 58 mm^44^) and lack obvious sexual dimorphism^52,53^; although Garcia et al*.*^54^ suggests they are sexually dimorphic in appearance in the ultraviolet region of the spectrum. Displays are performed by both sexes and triggered when another individual (even other species of lizards) appears in their field of vision^53^. The 3D model used herein was based on morphology and display footage taken from a population that inhabit dense clumps of spinifiex grass (*Triodia* sp*.*) and Mallee eucalyptus (*Eucalyptus* sp.) in the Murray-Sunset National Park, Victoria (Fig. S3c).

Both *G. longirostris* and *C. decresii* are highly territorial species that inhabit semi-arid rocky outcrops with low vegetation density. *Gowidon longirostris* are relatively large lizards (SVL 114 mm) mainly due to their exceedingly long tail compared to the other three species. The species is widely distributed in arid zones across western and central Australia^44^. The complex signalling sequence comprises limb waving and head bobbing, with push-ups followed by a forward body thrust/rise usually associated with tail lashing motions^55^. Throat and crest extensions are also common modifiers of the display (Fig. 1b). The model lizard used herein was based on morphology and display footage taken from a population located within and around Ormiston Gorge, within West-MacDonnell National Park, Western Australia. The habitat around Ormiston Gorge features large rocky outcrops with sparsely distributed vegetation including Eucalyptus trees, low shrubs (*Acacia* sp.) and a variety of native grasses and grass trees (*Xanthorrhoea* sp*.* Fig. S3b).

*Ctenophorus decresii* are averaged sized lizards (SVL 82 mm^44^) found across eastern South Australia, including Kangaroo Island^56^. The species exhibits diverse colouration within and between populations^57^ and is known to show individual variation in their agonistic displays^58^. Other than throat/crest extension, limb waving and head bobbing, *C. decresii* also displays a distinctive hind-leg push up with the tail coiled in a horizontal direction^56,59^ (Fig. 1d). The hind-leg push up is used by males to reflect endurance and aggressiveness during territorial interactions^58^. The model lizard used herein was based on morphology and display footages of *C. decresii* lizards inhabiting the rocky outcrops of the Flinders Ranges National Park, South Australia. The habitat is prone to fire, so we have incorporated charred fallen branches and sparse vegetation coverage, featuring mainly saltbush (*Atriplex nummularia*) and spinifex grass (*Triodia* sp.), as well as sparsely distributed *Eucalyptus* sp. and black oaks (*Casurina* sp.; Fig. S3d).

### Creating and animating the habitats

The microhabitats were modelled using Autodesk Maya 2015 based on data collected from habitat surveys. As emphasised in Chouinard-Thuly et al*.*^60^, the simulations created herein are based on data from real environments but our intention was not to precisely recreate a specific site. We used the habitat of *A. muricatus* from earlier work^27,36^, while the three new habitats were created as close replicates of the actual habitats. Briefly, the habitat models were constructed in accordance with a library of reference images photographed at each site during November 2016 and December 2017. These included 360° panoramic photographs to capture the general layout of the habitat, while microhabitat detail was captured by photographing plants and substrates within close range of a known signalling site. The position of plants surrounding the signalling point were noted and spatial layout information was incorporated into each scene.

As Maya provides generic and not species-specific plant models, major plant species from our study sites, such as spinifex grass and eucalyptus trees, were individually modelled and integrated with precise wind controllers for setting the speed, intensity and frequency of plant movements. The wind controllers are set as a sliding bar to vary in the range [0, 1], resulting in increasing movement intensity. Deformation of plant models in response to simulated wind used built-in scripts within Maya, which are based on physically accurate equations^61^. By manipulating the numbers on the wind controller, we set the movements of modelled plants to visually match video recordings of actual plant movements in response to moderate wind conditions at each site. Video recordings of plants at each site were taken during periods with natural wind speed close to 0 m/s and plant movements were generated using a leaf blower positioned 2 and 3 m from the plant, with wind speeds measured at the plant of 2.5 and 4 m/s, respectively. The camera was positioned 2 m from the base of the target plant and angled perpendicular to the direction of the wind emanating from the leaf blower.

### 3D lizard models and display sequences

Detailed steps on how 3D models of lizards were created are described elsewhere^27^. Lizard display sequences were created using video recordings of each species performing movement-based displays in nature and used rotoscoping techniques to match model movements with the footage upon which it was based. As described above, the displays of these four species are quite distinct. The temporal structure of the display is presented in Fig. 1, represented as the extent of body movement over time. A gradient detector model of computational motion analysis was used to detect motion in the image sequence^10^, and the number of non-zero cells were summed to provide the spatial extent of movement. The head-bob display of *C. fordi* generates the least movement (Fig. 1b), although relatively continuous throughout the sequence. In contrast, the display of *A. muricatus* is characterised by intermittent movement (Fig. 1a), beginning with small movements at the tip of the tail, then utilising the whole tail before the push-up component of the display. The temporal structure of both *G. longirostris* and *C. decresii* involve relatively constant movement once the display begins (Fig. 1c,d, respectively). The display of *C. decresii* involves a gradual increase in the amount of body movement before it ends, while *G. longirostris* incorporates several rapid changes in body movement (rapid increase and decrease). All lizard and habitat models were built in proportion and to scale.

### Scene composition and analysis

**Figure 2:**
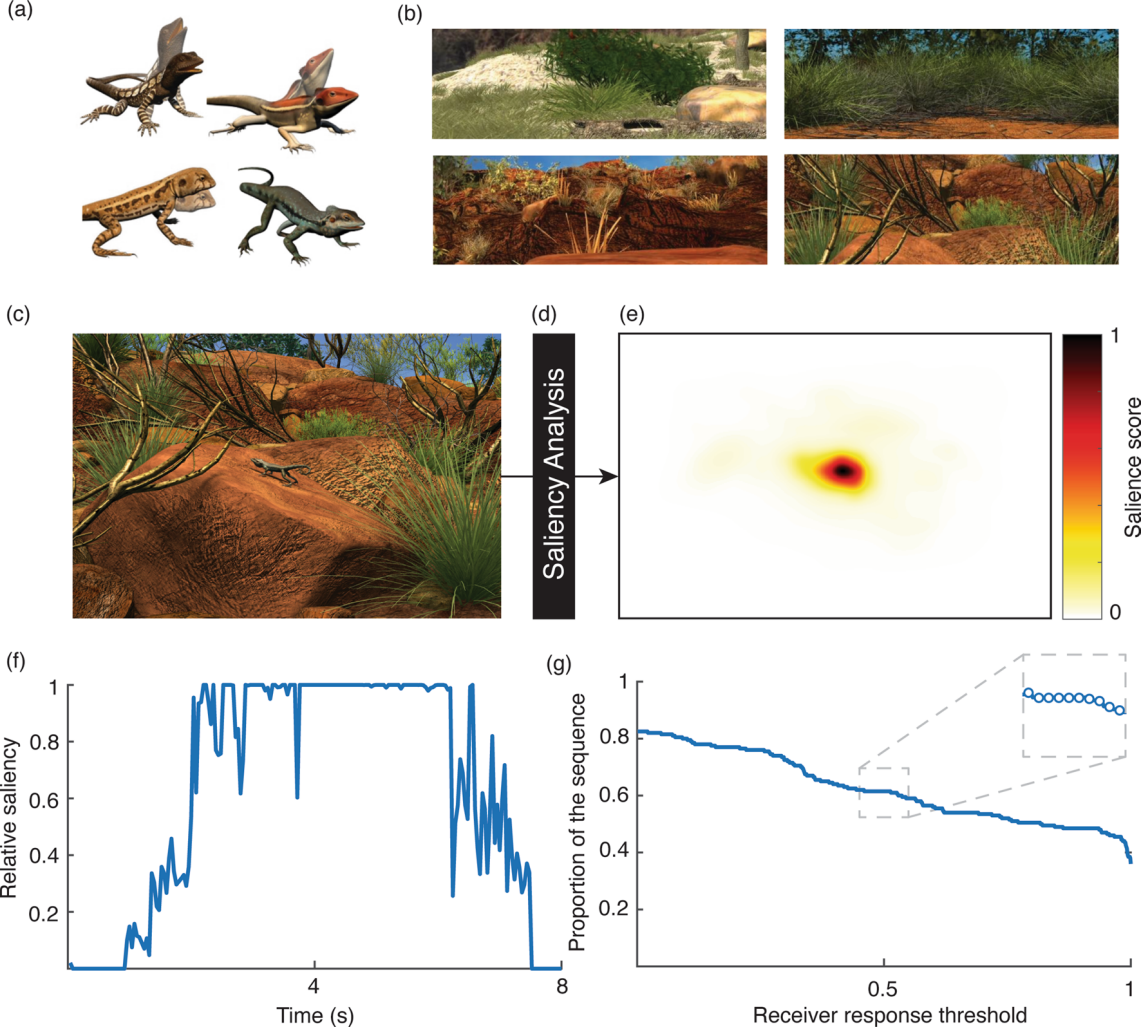
To examine the relative effectiveness of movement-based animal signals we created 3D animations of the displays of four Australian dragon lizards (**a**) and their respective habitats (**b**). Each species was inserted into each habitat (**c**) under varying environmental conditions. Animation sequences were then subjected to saliency analysis (**d**) to compute salience scores for all parts of the scene (**e**). Salience is computed in the range [0,1] and in this example corresponds to the position of the lizard in (**c**). The maximum salience score in the area of the scene featuring the lizard is identified for all frames in the animation sequence (**f**). We assumed the level of salience required to capture attention will vary within and between receivers and determined the proportion of the sequence that exceeded a given threshold as a function of varying receiver response thresholds (**g**). The performance vector comprises discrete scores at small increments in threshold values (**g**, inset). Lizard and habitat images were created by the authors using Autodesk Maya 2015 (https://www.autodesk.com.au/products/maya/).

The models of all lizard species were placed into the habitat of all species (Fig. 2a,b; Fig. S4). For a given habitat, the lighting conditions, location and orientation of the lizard in the scene and the position of the camera were matched for all lizard species. We used a single directional light to illuminate the entire scene with a uniform lighting effect. Lizard models were positioned in a given habitat according to where the local species would typically be found and orientated side-on to the camera. The animated signalling sequences of all lizard displays started at the same frame in the animation timeline, and were similar in duration (6.4 s or 160 frames, with approximately 5–10 frames differences between the set). All sequences were 8 s duration and exported as images at 25 fps and 720 × 576 resolution. We manipulated wind conditions by systematically increasing the extent of plant motion at all sites by manually adjusting plant controllers, confirming incremental (but non-linear) increases in noise using computational motion analysis as described in^36^. We repeated this to achieve ten versions of each scene varying in environmental noise conditions. All four species were placed in the habitats of each species under all ten noise conditions resulting in a final set of 160 sequences. For each species-habitat combination, we also created a baseline sequence featuring only lizard movements that is required for our analytical approach. Computational motion analysis^10^ was used to detect motion in the sequence and a binary mask was generated for each frame by converting all non-zero values to 1 to indicate where motion was detected.

Our analytical approach to determining salience of lizard displays was the same as previous work^27,36^. Briefly, animation sequences featuring plant movements were analysed frame by frame using saliency analysis. We used a graph-based model of visual saliency that incorporates local computations as well as global information in determining saliency maps^41^, and used only the motion channel. Here, motion is computed from an intensity version of the image using orientation selective Gabor filters, which reflects the presence of orientation-selective neurons in biological vision systems. Gabor filters tuned to four different orientations (0°, 45°, 90°, 135°) at nine spatial scales yields four Gabor pyramids. The inclusion of multiple spatial scales ensures that a wide range of velocities can be detected and is achieved by low-pass filtering and subsampling, decimating the input by a factor of two each time (1:1 to 1:256). Differences between Gabor pyramids are computed between successive frames using the Reichardt model for motion computation to yield motion pyramids (see^62^ and references therein for full mathematical descriptions), which are the sole contributor to saliency maps in our implementation (all other channels were turned off). We used these saliency maps to represent relative salience scores in the range [0, 1] for all areas of the frame (Fig. 2c–e). The scores of each sequence were then multiplied by its relevant binary mask, which featured only the lizard movement, to produce a relative salience score for the lizard movements in the environmental context imposed. This was repeated for all frames in the sequence to generate the relative salience over time (Fig. 2f). As in our earlier work, we then computed the proportion of the sequence that exceeded a given threshold as a function of changing receiver thresholds (1000 values between 0 and 1 varied at increments of 0.01; Fig. 2g). A single vector of values is obtained for each species in each wind condition of each habitat.

### Data analysis

Our objective was to compare and contrast the performance of lizard displays in different habitats under varying environmental conditions. We have simulated a single exemplar of each species and habitat to achieve this, so inferential statistics are not appropriate. Nevertheless, our simulations provide novel and informative data that we have considered quantitatively. We began by running non-metric multidimensional scaling (MDS) analysis, and used the *metaMDS* function from the *Vegan* package^63^ in the R Statistical Environment^64^ to quantify similarities/dissimilarities in the resultant vectors for each combination of species, habitat and wind condition (160 vectors). All data were considered together; however, to facilitate visual inspection of results, we group the outcome by wind condition and present the same results twice, labelled by the signalling species and then by habitat. Our second approach was to consider performance not as a single vector, but after segmenting individual datapoints (Fig. 2g inset) and grouping into three receiver threshold categories: low (0–0.3), moderate (0.3–0.8), high (0.8–1.0). We first confirmed the detrimental effect of wind for *A. muricatus* in its own habitat, before focusing on moderate receiver thresholds to compare species in all habitats during moderate and strong wind conditions.

### Ethical note

This study was conducted following ASAB/ABS’s Guidelines for the treatment of animals in behavioural research and teaching. Fieldwork undertaken to film lizard displays was completed with approval from La Trobe University’s Animal Ethics Committee (AEC 16-59) and under permits from Australian state government authorities: DEWLP Wildlife Research Permit No. 10008006 (Victoria), DEWNR Wildlife Permit No. U26541 (South Australia) and Parks and Wildlife Commission Permit No. 59056 (Northern Territory).

## Results

**Figure 3:**
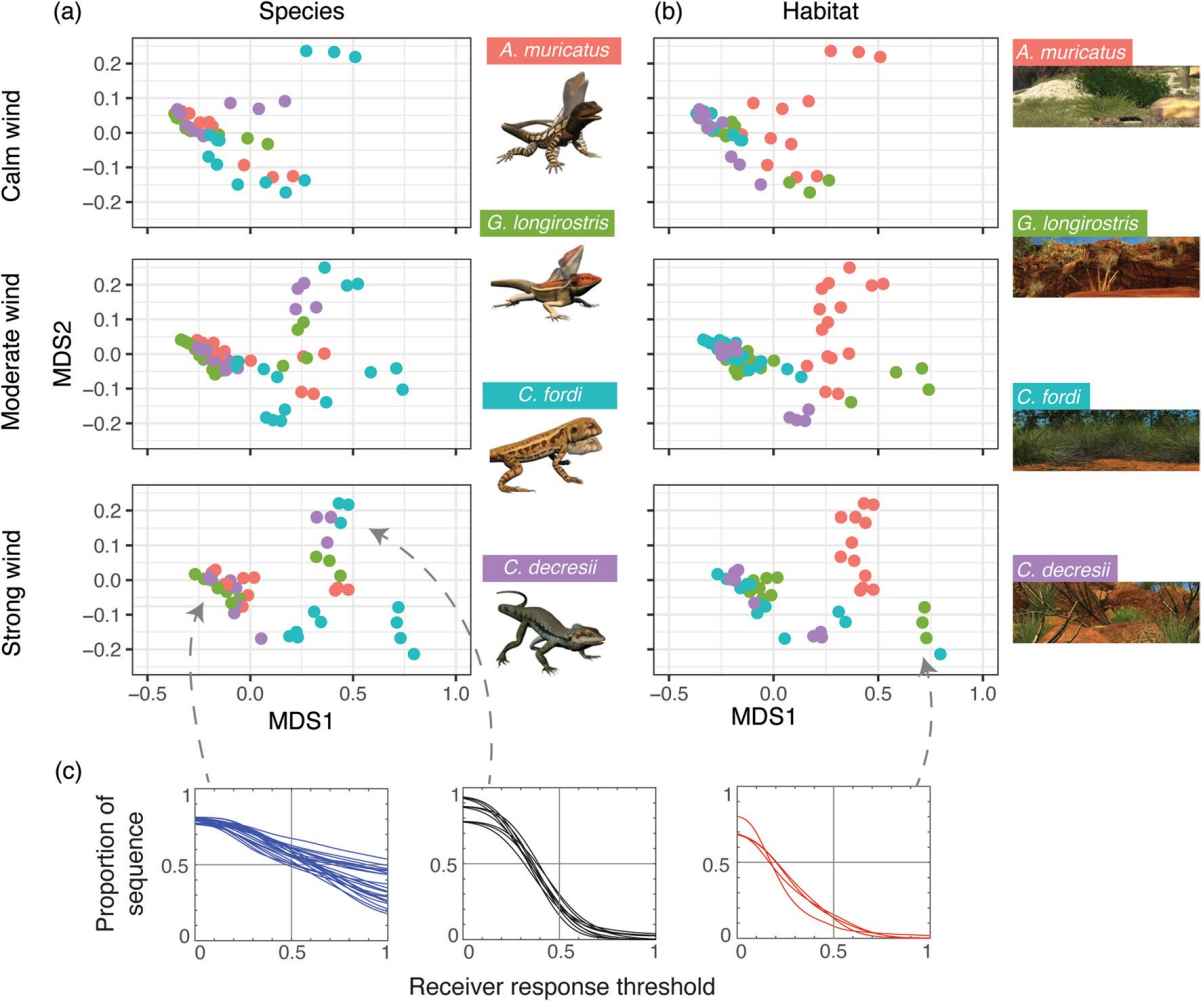
Results of non-metric multidimensional scaling analysis to compare similarities/dissimilarities between vectors summarising performance of lizard displays in multiple habitats under varying wind conditions. The results are shown twice, labelling data points by the lizard species (**a**) and by the habitat in which the lizard was placed (**b**). Separate plots show performance in calm (top row), moderate (middle row) and strong (bottom row) wind conditions. (**c**) To provide insight into the different clustering within MDS space, individual performance vectors that were used for MDS analysis are shown in strong wind for the best performing (blue lines), as well as poorer performing (black and red lines) display species-habitat combinations. Lizard and habitat images were created by the authors using Autodesk Maya 2015 (https://www.autodesk.com.au/products/maya/).

The effectiveness of displays by four Australian agamid lizard species were assessed in their own habitats and the habitats of each of the other species under different wind conditions. In each case, we obtained the proportion of the sequence that exceeded a specified threshold for response as a function of varying threshold values. Figure 3 provides the result of non-metric MDS analysis comparing similarities/dissimilarities between all 160 resultant vectors. The XY coordinate data are presented separately for calm (levels 1–3), moderate (levels 4–7) and strong (levels 8–10) wind conditions. With datapoints labelled by the species displaying (Fig. 3a), we observe some grouping by species, though these appear in clusters of a few points (i.e., a single habitat) rather than as a single species cluster that would imply similar performance across all habitats and conditions. Another observation we can make here is that the performance of three species (*A. muricatus, G. longirostris* and *C. decresii*) are comparable in certain habitats (Fig. 3c, blue lines), while one species, *C. fordi*, is consistently on the outer and performs relatively poorly (Fig. 3c, red lines). In Fig. 3b we label data by the habitat in which displays are performed. What is most striking here is that displays by all species within the habitat of *A. muricatus* cluster together, and this gets tighter as wind conditions worsen. Performance in this habitat is strong to begin with, but declines as receivers become more discriminating (Fig. 3c, black lines), suggesting that the task of distinguishing signal from noise has become more difficult.

**Figure 4:**
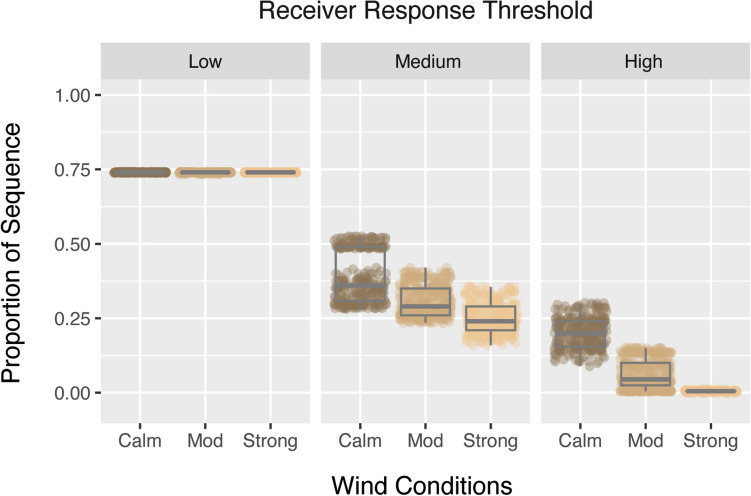
The performance of A. muricatus in its own habitat as a function of wind conditions and receiver response threshold. The y-axis indicates the proportion of the sequence exceeding a given threshold, with separate plots for low (0–0.3), medium (0.3–0.8) and high (0.8–1) thresholds and wind conditions on the x-axis.

**Figure 5:**
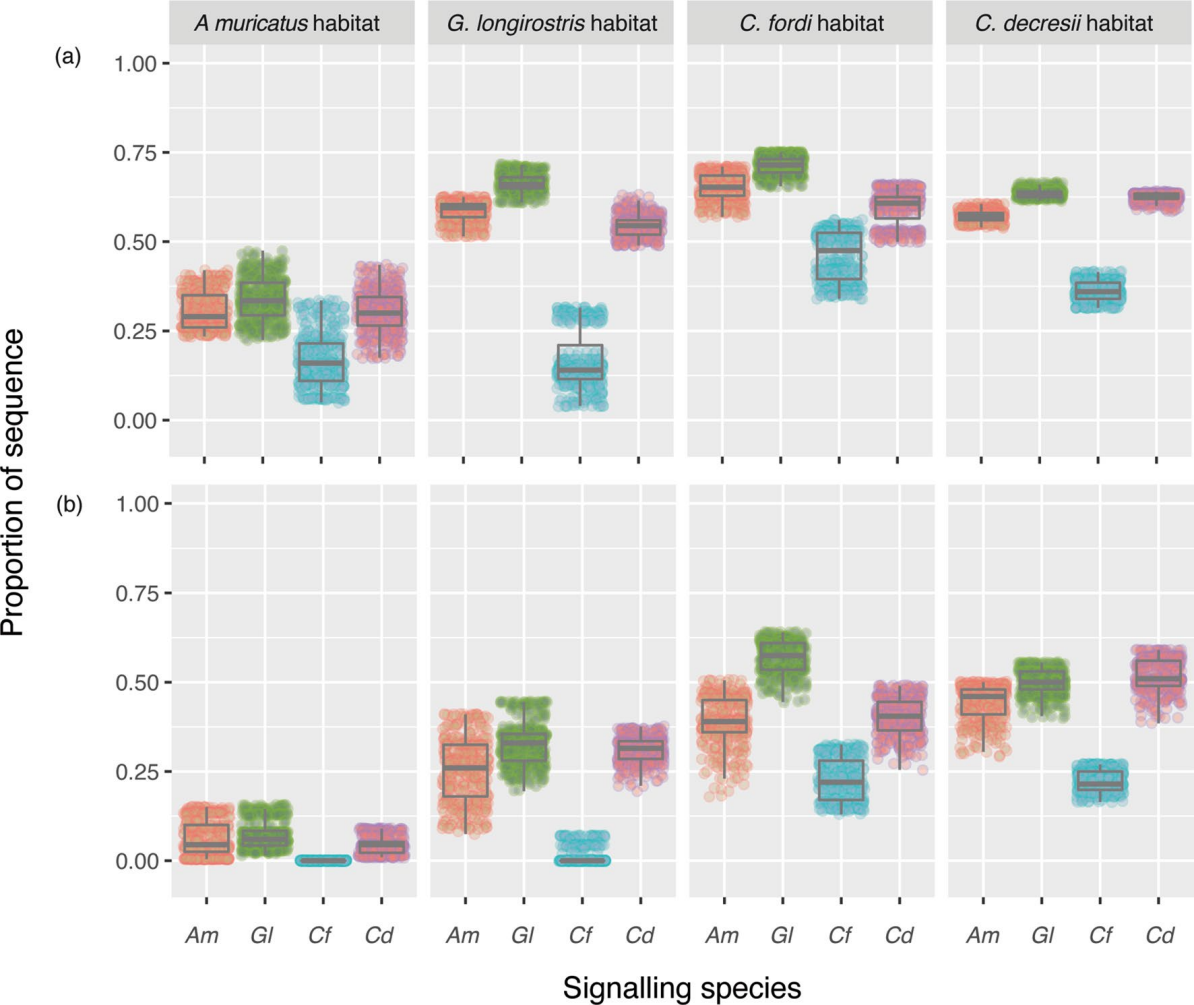
The relative performance of lizard displays under moderate (**a**) and strong (**b**) wind conditions with medium receiver response thresholds (0.3–0.8). Separate plots are showing for the habitats in which signalling takes place, with each showing the proportion of the sequence exceeding the threshold shown as a function of signalling species. (Am—*A. muricatus*, GL—*G. longirostris*, Cf—*C. fordi*, Cd—*C. decresii*).

We next considered the data as individual performance scores grouped into low, moderate and high receiver threshold categories. Figure 4 provides the outcome for *A. muricatus* in its own habitat. When receivers are non-discriminating, display performance is strong even as wind conditions increase. However, the masking effect of wind becomes apparent when receivers start to be more selective in what will attract attention. Given highly selective receivers are predicted to rarely detect a signal in strong wind conditions, we chose to focus on moderate receiver response thresholds to compare species across all habitats. The performance of each species’ display in each habitat is shown in Fig. 5 for moderate and strong wind conditions (Fig. 5). These data confirm our earlier observations that the habitat of *A. muricatus* is the most detrimental to display performance, while the display of *C. fordi* is consistently the worst performing. We also see that stronger wind reduces performance across all sites (scores reduced in Fig. 5b compared with Fig. 5a), although to varying degrees (greater spread in data in strong winds).

## Discussion

Technical difficulties in quantifying environmental conditions and movement-based displays in a systematic way has made examination of movement-based signals a challenging task^65^. Legislative restrictions have made in situ cross-habitat comparisons improbable. Recent studies have attempted to contrast multiple signals in multiple habitats by quantifying signal and noise separately and then contrasting resultant motion speed data^33,34^. For the first time, sophisticated 3D animations gave us the opportunity to closely examine movement-based signals in multiple habitats. Specifically, we were able to quantify how varying environmental conditions, receiver behaviour and differences in display and microhabitat structures effected signal effectiveness. Using four Australian agamid lizard species, we compared and contrasted how species-specific movement-based signals would perform in structurally distinct environments. Our simulations suggested that signal effectiveness varies as a function of receiver response thresholds, where at medium to high thresholds, the changes within and between species became clear. In line with our predictions, all signals performed the worst in *A. muricatus* habitat, the performance of *G. longirostris* and *C. decresii* were very similar and the display of *C. fordi* was the worst across all habitats. However, contrary to our prediction, the performance of *A. muricatus* was not more effective than *G. longirostris* and *C. decresii*, so the notion that signals expected to be adapted to a noisier environment will perform better than the ones adapted to less noisy environments was not supported.

The poor performance of the display by *C. fordi* relative to other species was anticipated and might be attributable to differences in signal function. Unlike the other three species in the study, *C. fordi* are not territorial and their displays are not considered aggressive responses to intruders. It has been suggested that the likely function of the simple head bob display is for species recognition^53^. Displays of this species are performed at close range when lizards encounter other lizards, so the necessity to distinguish itself from surrounding plant motion is removed. The other three species in this study are all assumed to be territorial species^50,59,66^, responding to intruders at greater distances and when the intruder is possibly unaware of the presence of the territory owner. In these circumstances, reliable detection and processing of territorial displays are essential for establishing social dominance^67,68^.

The potential masking effect for movement-based signals is dependent on prevailing wind and the distribution of plants in the environment^32^. Complex image motion backgrounds are expected in dense vegetation, which would likely require animals to generate certain types of movements for effective communication^3^. The habitat of *A. muricatus* is densely vegetated with tall grasses and small shrubs (Fig. S1a). The plants are often positioned close to the signalling animal, with short signaller-plant distances predicted to reduce detectability of signals^69^. In comparison, the habitats of *G. longirostris* and *C. decresii* feature sparsely vegetated environments, with plants typically separated from the signalling animals. However, the *C. decresii* habitat we modelled herein was based on a recently burnt area which would feature a reduced level of motion noise in the environment, this information could change for *C. decresii* as the vegetation recovers from the fire over the next few years. The signaller-plant distances in the habitat of *C. fordi* were also very close, but in this case the dominant plant species, spinifex grass (*Triodia* sp.), does not generate considerable movement in response to wind at the base of the plant where the lizards were positioned. As such, the potential masking effect of plant environments is attributable to the interaction between wind conditions, the spatial position of plants in the environment and the structure of the plant itself that will dictate how it responds to wind.

Habitats with common structural properties, regardless of geographic location, are predicted to result in signals with similar structural characteristics^70,71^. Both *G. longirostris* and *C. decresii* inhabit sparsely vegetated rocky outcrops, and the similarities in the structure of their signals and relative signal performance in noise could be attributed to structural similarities in the environments they inhabit. Interestingly, the performance of their displays was just as effective as the signal of *A. muricatus* in the habitat of *A. muricatus* suggesting different movements might be equally effective. However, it is in the other habitats that we start to see subtle interactions between display and habitat structure. The displays of *G. longirostris* perform best in two of these habitats, while *C. decresii* displays perform better in their own habitat. Ramos and Peters^55^ found no evidence for broad scale habitat characteristics influencing signal use in Australian dragons, but it is intriguing to consider the possibility that the use of different motor patterns is partially driven by fine-scale habitat characteristics that influence the motion noise environment. Both *G. longirostris* and *C. decresii* signals involve almost continuous movement featuring whole body push-ups/forward thrusts. In contrast, the push-up component of the display of *A. muricatus* is brief and follows an occasionally lengthy period of tail flicking^14^. Tail-flicks represent the introductory component to the rest of the display, reportedly to capture the attention of a receiver^14,51^. It is also this component of the signal that exhibits plasticity in response to changing environmental conditions^14^, whereby the duration of tail flicking is lengthened in noisy conditions and the movement becomes more intermittent. Tail-flicks are observed in *C. decresii,* although it is not a core component of the display^35^, and there are no reports for tail flicking by *G. longirostris*^55^*.* Further research is needed to determine whether either of these species possess a component of their display that exhibits the kind of plasticity needed for variable environments. Alternatively, perhaps the environments that they inhabit have not selected for such plasticity as the rocky gorges provide a clear background against which the signal can be reliably detected regardless of plant movement speeds.

This study has added to a growing literature showing that habitat characteristics and the associated noise landscape are relevant to signal design and likely promotes changes in signalling strategies under certain conditions^14,72,73^. How signals function in social interactions is another important consideration for driving variation in signal structure^53,67,74,75^. Cross-habitat examination of signal performance has been beneficial for other communication systems^19–21,23^, but the present study is one of the first comparable studies for movement-based signals. Our innovative approach allowed for examination of different movement-based signals embedded in noise across different habitats. We acknowledge that the results herein are generated from a single representative display per species, in a single representative habitat per species. Nevertheless, our method and analytical approach has provided quantitative data showing that the effectiveness of movement-based animal signals will be affected by the motion noise environment, and might indeed direct signalling behaviour in much the same way that it has for other modes of signalling.

## Supplementary Information

Supplementary Figures.
