# Supplementary material for: Simulations with Australian dragon lizards suggest movement-based signal effectiveness is dependent on display structure and environmental conditions
Source: Sci Rep. 2021 Mar 18;11:6383. doi: 10.1038/s41598-021-85793-3 (PMC7973430; doi:10.1038/s41598-021-85793-3)
Supplement: Supplementary file 1 — Supplementary Figures. [file 41598_2021_85793_MOESM1_ESM.pdf]

Supplementary material accompanying

# Simulations with Australian dragon lizards suggest movement-based signal effectiveness is dependent on display structure and environmental conditions


Xue Bian[1], Angela Pinilla[2], Tom Chandler[2], Richard Peters[1*]

[1]Animal Behaviour Group
Department of Ecology, Environment and Evolution
La Trobe University
Melbourne, VIC, Australia

[2]Faculty of Information Technology
Monash University
Caulfield East, VIC, Australia

*Correspondence:
Dr. Richard Peters
Email: richard.peters@latrobe.edu.au


**Figure S1** | Species and habitats used in the present paper. *Left column:* the typical signalling sites at which the target species are usually seen. *Right column:* general habitat structure and representative plants found in the habitats of all of the study species. (a) *Amphibolurus muricatus* are usually found basking on fallen wood. The habitat is densely vegetated with tall grasses and shrubs. (b) *Gowidon longirostris* inhabit arid rocky outcrops with sparsely vegetated eucalyptus and low shrubs, and are often seen displaying on rocks. (c) *Ctenophorus fordi* are small lizards that can be found between dense spinifex grass clumps in dry sandy areas of Victoria. (d) *Ctenohorus decresii* are colourful lizards that inhabit rocky ranges with typical vegetation of spinifex grass and eucalyptus, and is often seen displaying on rock surfaces. Images of lizards in (c) and (d) by Jose Ramos.

*Figure on following page…*

(a)

(b)

(c)

(d)

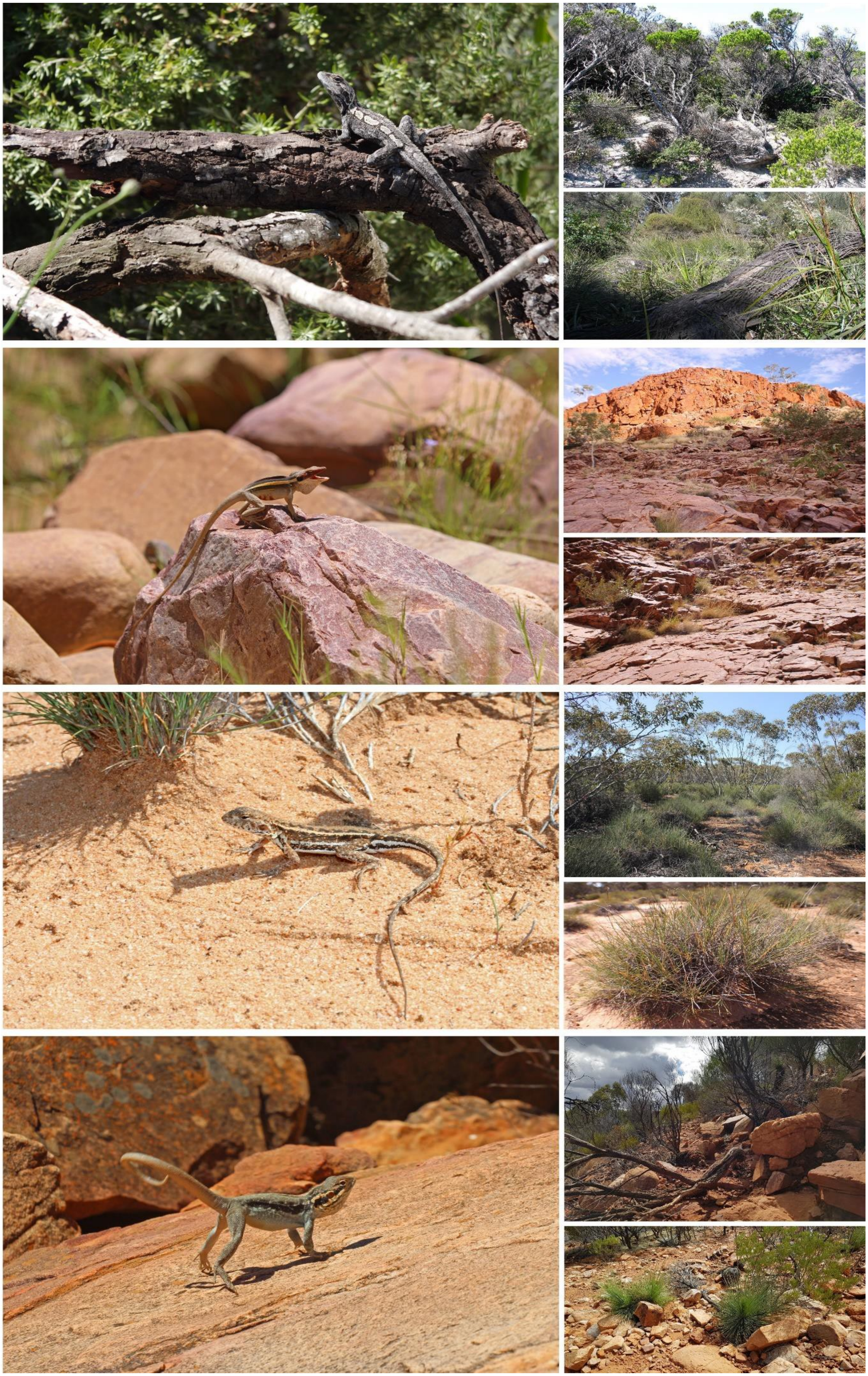

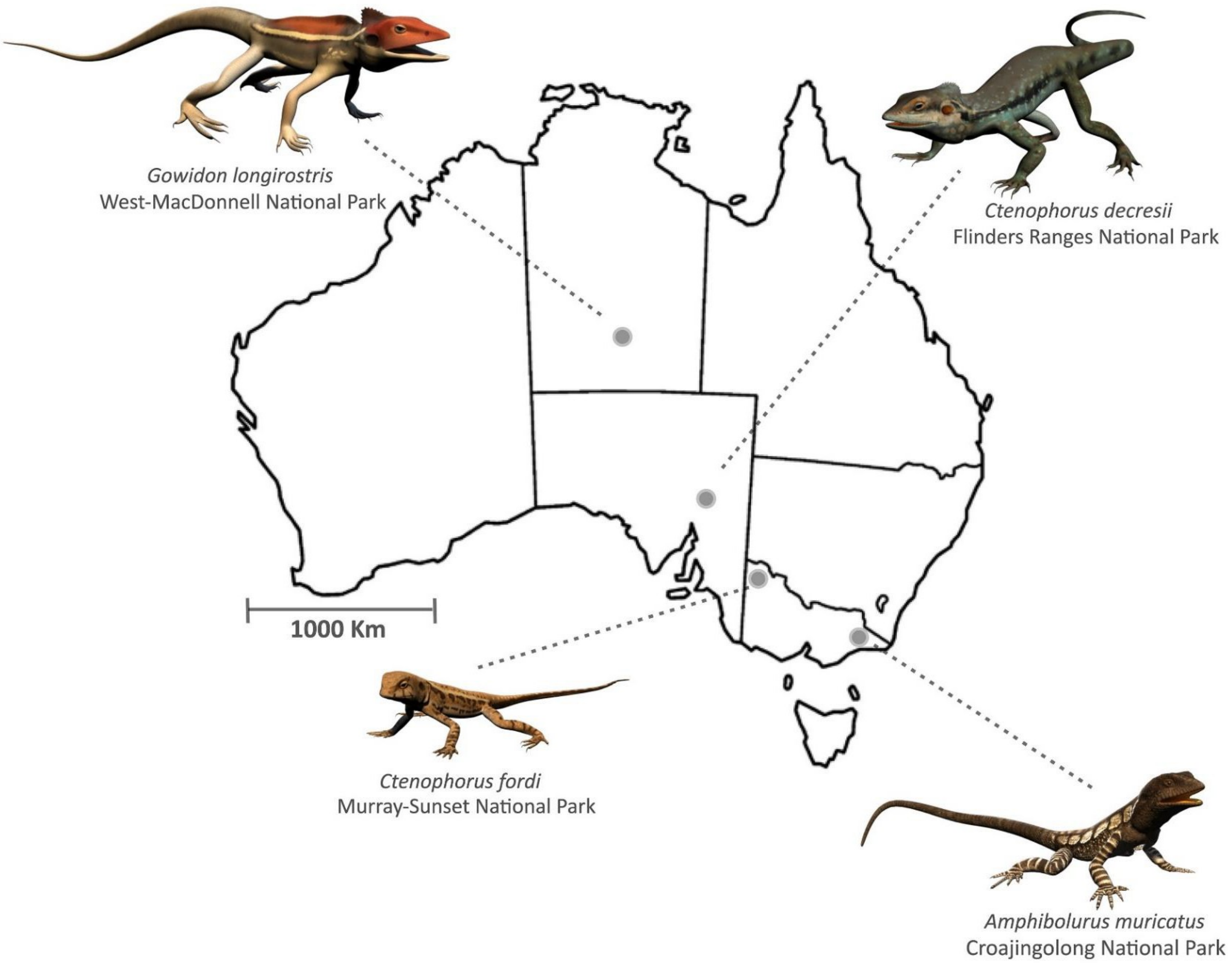


**Figure S2** | Models of target species and sampling sites in Australia. Lizard images were created by the authors using Autodesk Maya 2015 (https://www.autodesk.com.au/products/maya/).

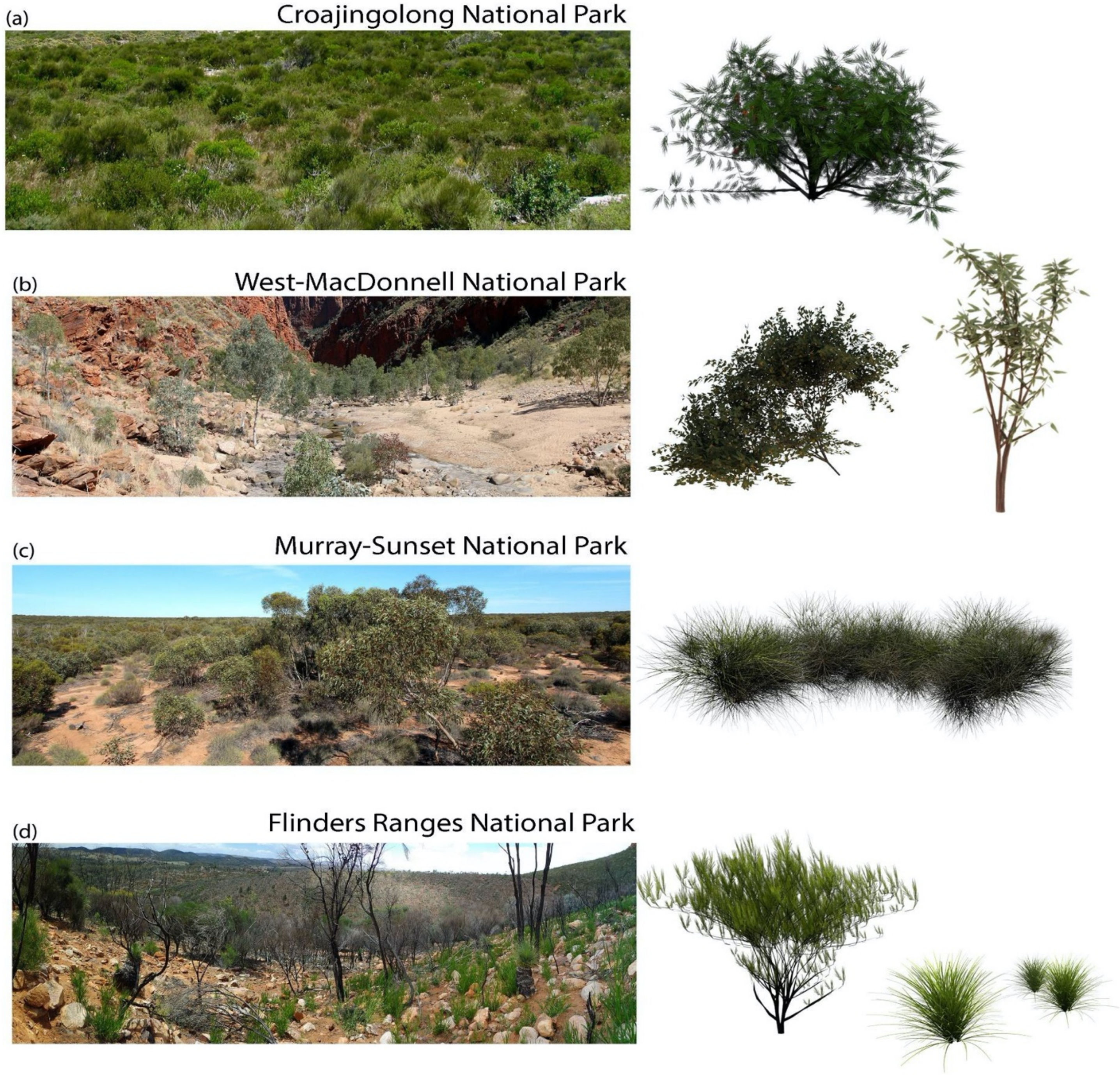


**Figure S3 |** Study sites of the focal species (*left*) and 3D models of typical plant species featured in each habitat (*right*). (a) Habitat of A*mphibolurus muricatus* at the Croajinglong National Park is densely vegetated with grasses and typically featuring *Grevillea* sp. (b) Habitat of *Gowidon longirostris* at West-MacDonnell National Park, usually features low shrubs (*Acacias* sp.) and Eucalyptus. (c) Habitat of *Ctenophorus fordi* at Murray -Sunset National Park that typically featuring dense clumps of spinifiex grass (*Triodia sp.*) and Mallee eucalyptus (*Eucalyptus* sp.). (d) Habitat of *Ctenophorus decresii* at the Flinders Ranges National Park, this habitat features saltbush (*Atriplex nummularia*) and spinifex grass (*Triodia* sp.). Habitat photos in (b)-(d) by Jose Ramos. Plant models created for this project by Xue Bian and Angela Pinalla

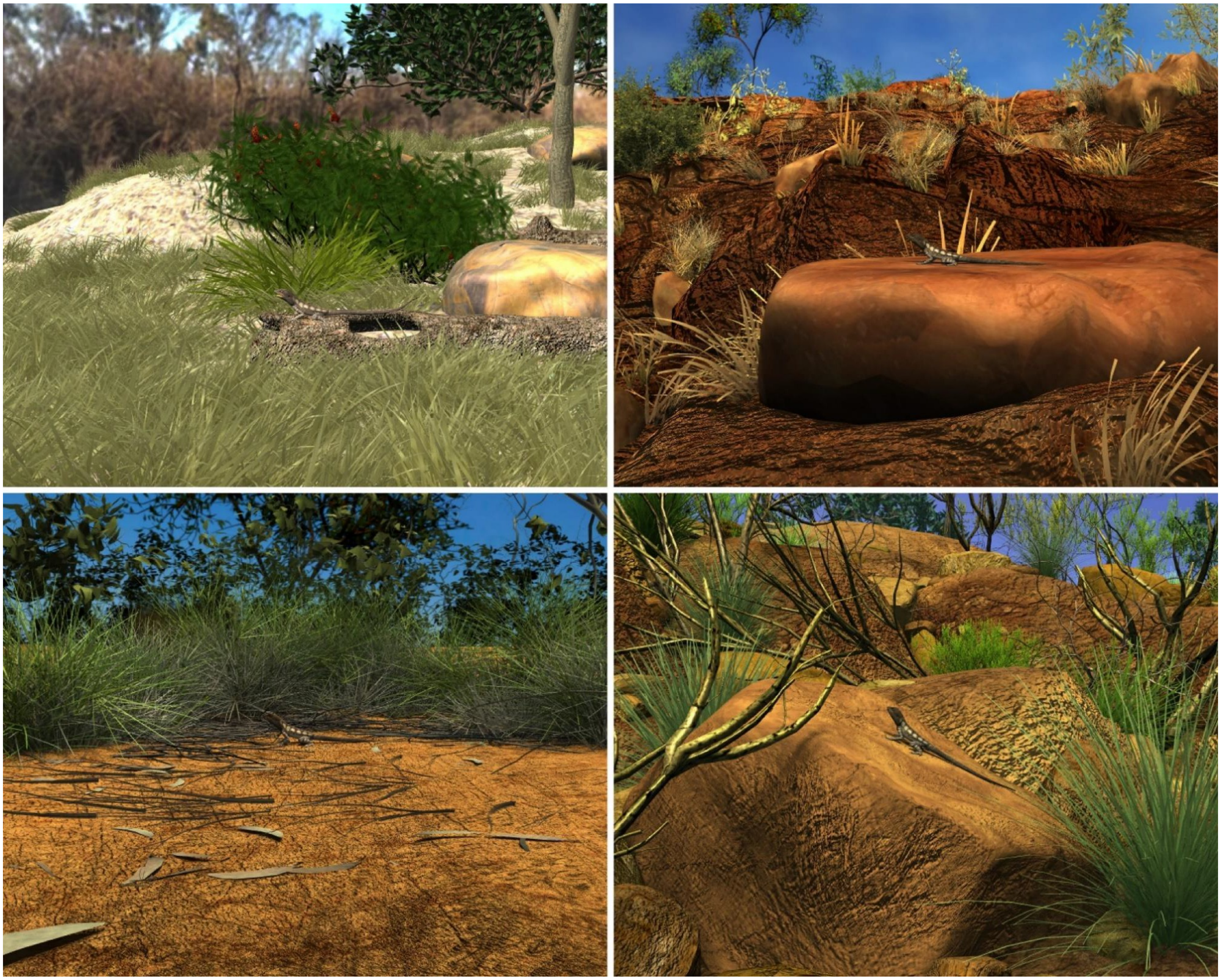

**Figure S4 |** (a) High resolution renders of one representative frame showing *A. muricatus* in all four habitats. Habitats are (clockwise from top-left): *A. muricatus, G. longirostrisi, C. decresii* and *C. fordi*. Images of lizards in habitats were created by the authors using Autodesk Maya 2015 (https://www.autodesk.com.au/products/maya/).
